# Compartment Syndrome: An Uncommon Twist in Childhood Henoch-Schönlein Purpura

**DOI:** 10.7759/cureus.63462

**Published:** 2024-06-29

**Authors:** Nadia Bouhafs, Aziza Elouali, Chaimae N'joumi, Kamal El Haissoufi, Mohamed Belahcen, Maria Rkain, Abdeladim Babakhouya

**Affiliations:** 1 Pediatrics, Centre Hospitalier Universitaire Mohammed VI Oujda, Oujda, MAR; 2 Faculty of Medicine and Pharmacy, Mohammed First University of Oujda, Oujda, MAR; 3 Pediatric Surgery, Mohammed VI University Hospital of Oujda, Oujda, MAR; 4 Pediatric Gastroenterology, Centre Hospitalier Universitaire Mohammed VI Oujda, Oujda, MAR; 5 Pediatrics, Mohammed VI University Hospital of Oujda, Oujda, MAR

**Keywords:** fasciotomy, surgical emergency, rare complication, compartment syndrome, henoch-schönlein purpura

## Abstract

Henoch-Schönlein purpura (HSP) also known as rheumatoid purpura is the most common vasculitis in children. This condition affects small blood vessels, predominantly targeting the skin, digestive system, joints, and kidneys. Short-term prognosis mainly depends on abdominal complications, while long-term prognosis is mainly determined by the severity of kidney involvement, which occurs in about 35% of cases. Although uncommon, other organs such as the lungs, heart, or nervous system may also be affected. Compartment syndrome of the hand and forearm is a very rare complication of HSP. To our knowledge, only two cases have been reported in the literature. We describe the case of a four-year-old child who presented with rheumatoid purpura complicated by compartment syndrome of the hand and forearm successfully managed through emergency fasciotomy.

## Introduction

Rheumatoid purpura stands as the most prevalent systemic vasculitis affecting children in the Western world. The first descriptions of the disease were made by Dr. Heberden in 1801 and further elaborated by Drs. Henoch and Schönlein in 1837, hence the name Henoch-Schönlein purpura (HSP) in some countries [1]. This IgA-mediated leukocytoclastic small vessel vasculitis primarily affects the skin, joints, digestive tract, and kidneys. Rheumatoid purpura usually follows a benign course, resolving spontaneously within approximately 10 days, visceral complications being rare.

In this article, we present a severe case of HSP complicated by gastrointestinal bleeding, scalp hematoma, IgA nephropathy, and compartment syndrome of the upper limb, the latter being an extremely rare complication necessitating urgent surgical intervention, requiring decompressive fasciotomy as soon as possible to mitigate potentially severe consequences.

## Case presentation

**Figure 1:**
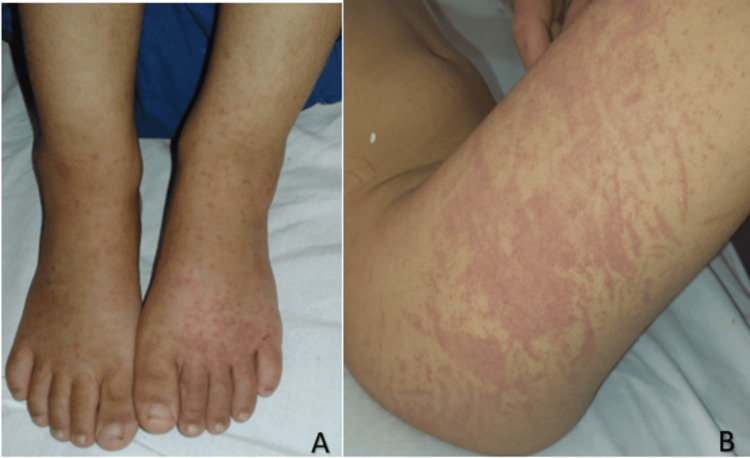
A. Image showing a vascular purpura with a slight edema of lower limbs. B. Clinical presentation of the child on the third day after his admission showing the purpura extension to the thigh.

A four-year-old boy, with no previous medical issues, was brought to the pediatric emergency department complaining of abdominal pain, inflammatory joint pain affecting large joints, and orthostatic purpura. The diagnosis of HSP was made based on the typical presentation. Within 48 hours, the purpura spread, and the abdominal pain and arthralgia worsened, prompting hospitalization in the pediatric ward for pain management. Upon admission, clinical examination revealed a child in significant pain, with altered general condition, normal blood pressure reading of 90/50 mmHg, and no fever, with a negative urine dipstick. In addition, a mild swelling of the ankles was observed (Figure 1). Initial evaluations showed thrombocytosis at 490,000/mm^3^ and an erythrocyte sedimentation rate (ESR) of 17 mm. Electrolyte levels, kidney function, and hemostasis tests were all within normal ranges. The progression was marked by worsening symptoms: extension and recurrence of purpura, increased joint pain, and reappearance of severe abdominal pain suggestive of intussusception. Given the severity of the symptoms, the patient was initiated on treatment with full-dose corticosteroids at 2 mg/kg/day.

**Figure 2:**
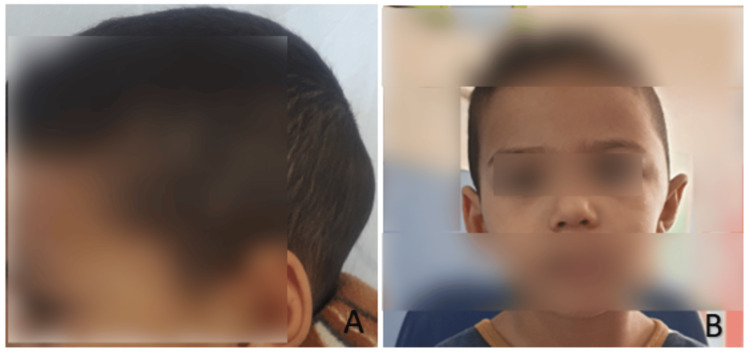
Clinical image of our patient four days after his admission showing the presence of scalp edema. Face (A)/profile(B).

**Figure 3:**
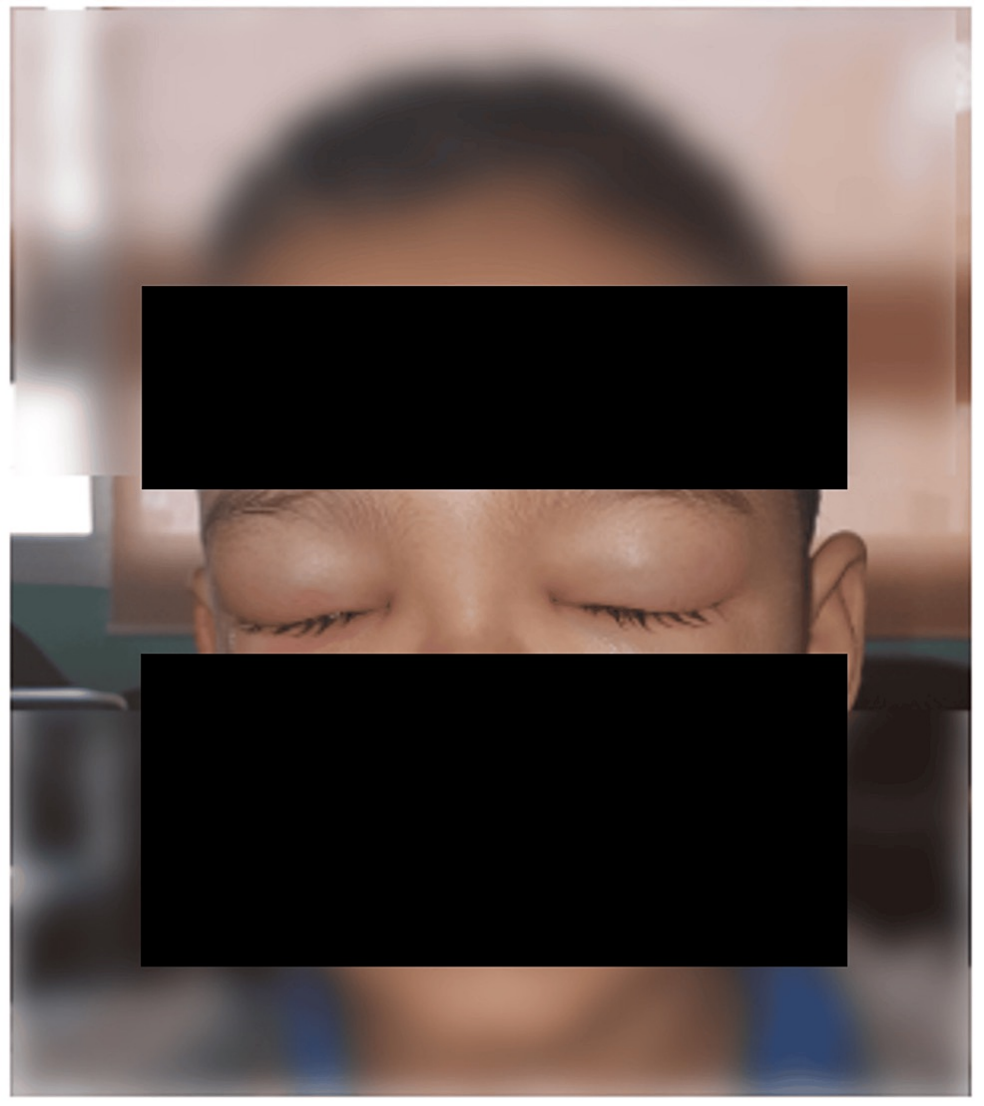
Clinical image of our patient six days after his admission showing the presence of eyelid swelling.

Four days after admission, the child developed scalp edema (Figure 2) and then bilateral eyelid swelling the day after (Figure 3). Blood pressure remained within normal range, while urine dipstick revealed proteinuria. Laboratory tests revealed 24-hour proteinuria of 4.1 mg/kg/day, with normal renal function and normal C3-C4 levels. Antinuclear antibodies were negative, but a significant decline in factor XIII to 28% was noted, often indicative of the risk of renal or digestive complications. Three days later, the patient experienced excruciating abdominal pain, followed by three episodes of rectal bleeding. Abdominal ultrasound revealed a hematoma of the intestinal wall. Due to the severity of the clinical presentation (severe digestive involvement), the patient was promptly started on corticosteroid bolus therapy at 30 mg/kg/day and intravenous immunoglobulin infusion at 1 g/kg/day.

**Figure 4:**
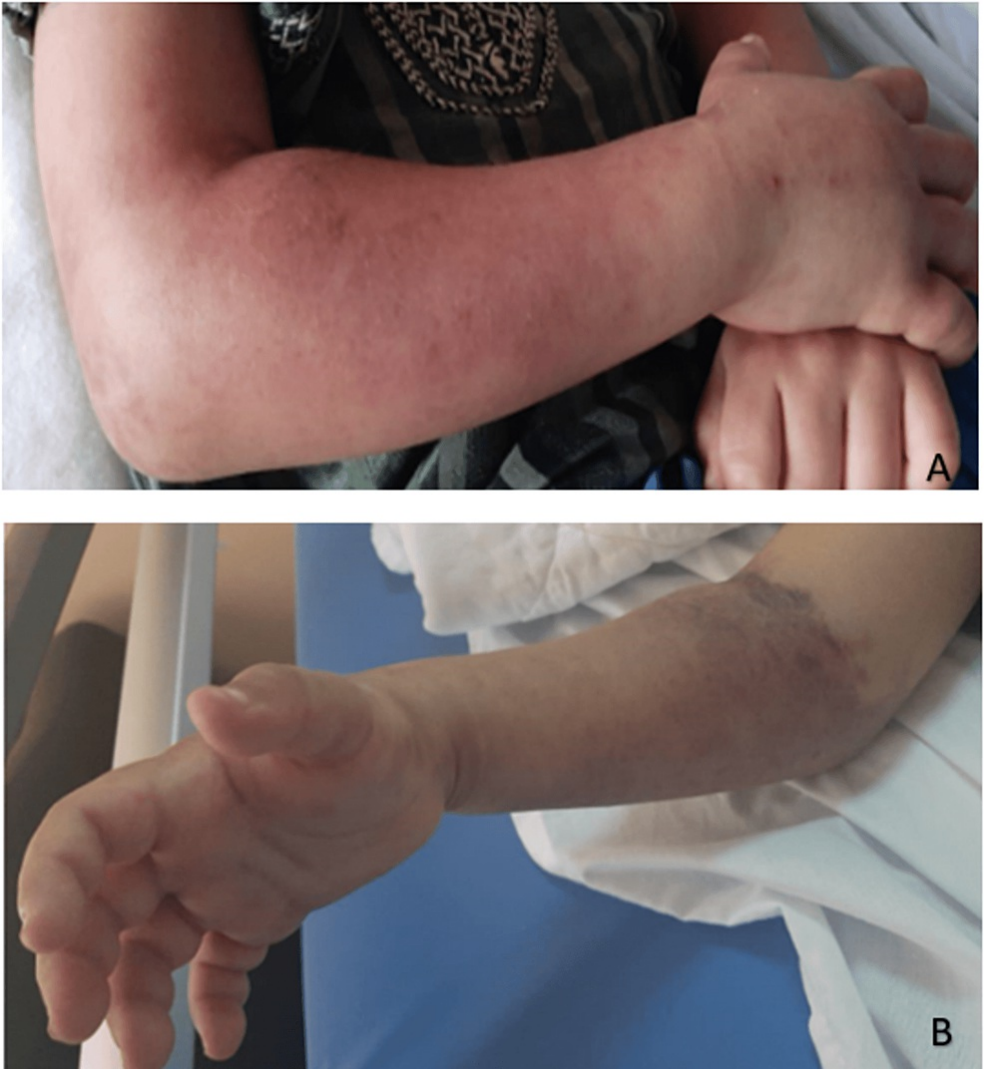
Anterior (A) and posterior (B) clinical views of the right hand and forearm of the child showing the occurrence of acute compartment syndrome after his admission where surgical treatment was urgently indicated.

**Figure 5:**
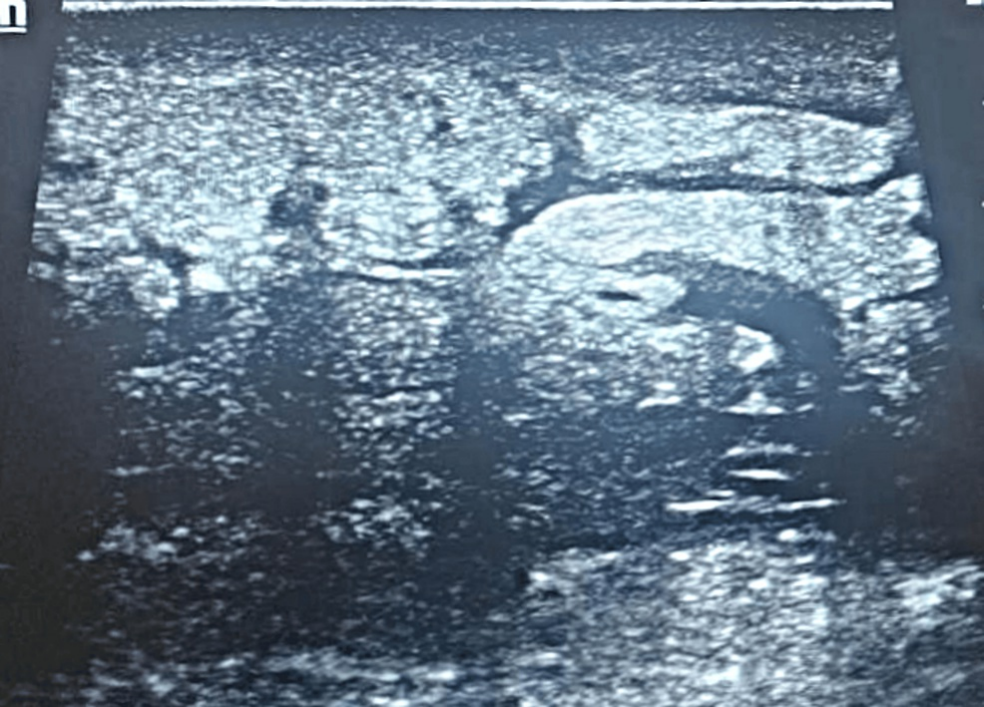
Emergency ultrasound image showing soft tissue infiltration.

**Figure 6:**
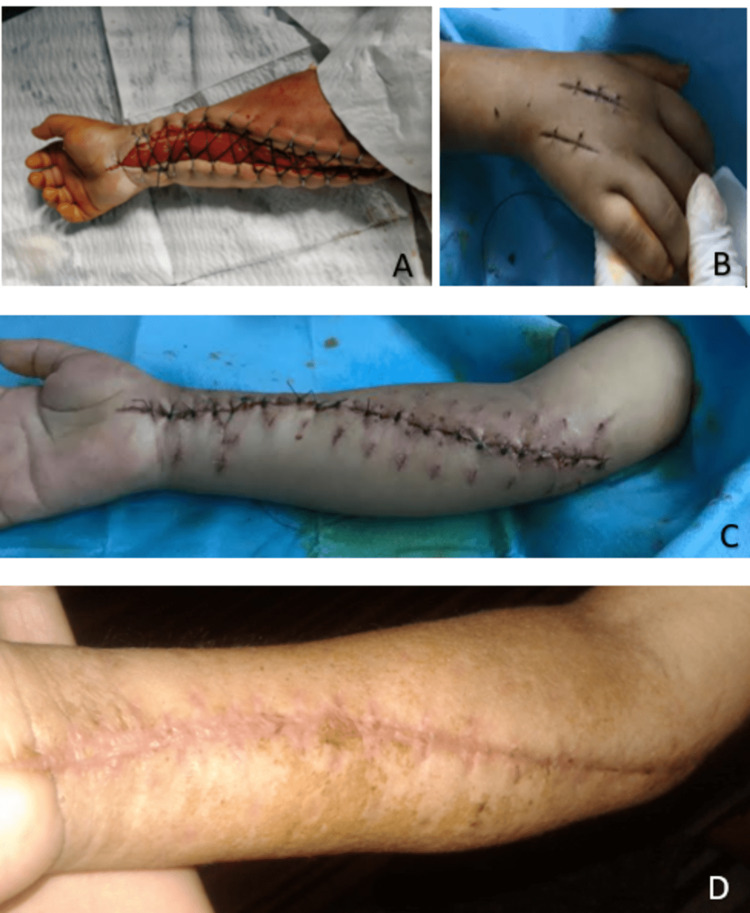
A: Intraoperative view showing the final aspect of the performed fasciotomy of the right forearm because of acute compartment syndrome. B, C: Intraoperative images a few days after surgery showing the final view after progressive and gradual fasciotomy closure without infectious tension signs. D: Clinical image of the anterior forearm of the child showing good wound healing three weeks after fasciotomy.

On the following day, around 11 am, the child began experiencing intense, tension-like pain in the right wrist, accompanied by local swelling that rapidly spread to the hand and forearm, extending downward to the lower arm. Clinical examination revealed a swollen limb with numerous purpuric spots, while pulses remained detectable and the skin showed signs of redness and warmth. Subsequent progression led to livid discoloration, indicating ischemia, along with flexion of the proximal interphalangeal joints (Figure 4). An emergency Doppler ultrasound was performed to rule out thromboembolic events, revealing soft tissue infiltration by the swelling (Figure 5). The radial and ulnar arteries and corresponding veins were patent. A diagnosis of compartment syndrome was established, warranting immediate transfer to the operating room at around 3 pm for a fasciotomy procedure. Intraoperatively, evidence of edema and hematoma within the flexor compartment was noted. Gradual approximation of wound edges was performed, resulting in satisfactory wound healing (Figure 6).

**Figure 7:**
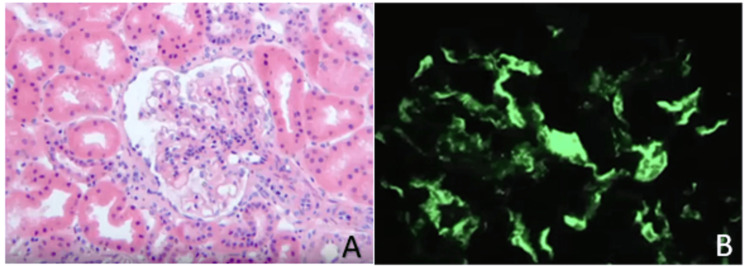
Photomicrograph of a renal biopsy shows a glomerulus with mild mesangial proliferation, without endocapillary proliferation or fibrosis (A: HE x1000). Immunofluorescence microscopy reveals IgA deposits in the mesangium (B).

A few days later, follow-up examinations revealed a significant increase in platelet count and elevated white blood cell count, while factor XIII levels remained low, indicative of IgA nephropathy. By day 17, proteinuria had surged beyond 100 mg/kg/day, accompanied by a decrease in albumin levels to 25 g/l. A renal biopsy confirmed the diagnosis of IgA nephropathy (Figure 7), prompting a three-month treatment regimen with cyclophosphamide. Encouragingly, the patient exhibited positive clinical and biochemical responses to the treatment.

The biological findings of the patient are presented in Table 1. 

**Table 1:** Biological findings of our patient.

Parameters	Laboratory tests upon admission	Four days after admission	17 days after admission	Normal values
Platelets (E/mmᶟ)	427000	521000	911000	150000-400000
White blood cells (E/mmᶟ)	12670	10050	17340	7000-10000
Sedimentation rate (mm)	17	-	41	<15
C-reactive protein (CRP) (mg/L)	15	-	3	6-10
Prothrombin rate (%)	89	-	-	70-100
Albumin (g/L)	36	31	25	35-45
Urea (g/L)	0.21	0.24	0.28	0.18-0.48
Creatinin (mg/L)	3.73	2.44	3.14	3.1-4.7
Complement C3 (g/L)	-	1.71	-	0.88-2.01
Proteinuria (mg/24H)	-	57	1481	<150
Factor XIII (%)	-	28	21	70-140

## Discussion

This marks the first recorded instance of upper limb compartment syndrome in the context of HSP within our institution, adding to the two other cases documented in existing literature. Complications such as severe gastrointestinal bleeding, pulmonary hemorrhage [2], intracranial hemorrhage [3], bilateral occlusion of the central retinal artery [4], orbital hematomas, penile involvement [5], thrombosis [6], appendicitis, hemorrhagic bullous lesions [7], myocarditis, and intracardiac thrombus [8] have been previously reported.

Rheumatoid purpura, also known as HSP, is a systemic vasculitis affecting small-caliber vessels, primarily associated with predominant immune deposits of immunoglobulin A (IgA) [9]. It stands as the most prevalent vasculitis in children, with an estimated annual incidence of around 10 cases per 100,000 individuals [10]. Typically regarded as a condition of early childhood, its peak occurrence falls between the ages of five and six years, mostly seen in males with a sex ratio between males and females of 1.9:1 [1]

In 25-90% of cases, a triggering factor is identified, most commonly bacterial infections (*Streptococcus *and* Mycoplasma*) or viral infections (parvovirus B19, Epstein-Barr virus (EBV), and varicella-zoster virus), less frequently parasitic infections and occasionally medication use, and, exceptionally, vaccination [11]. The diagnosis of HSP is primarily clinical, with complementary examinations providing limited contribution.

In 1990, the American College of Rheumatology proposed the following criteria to distinguish HSP from other forms of vasculitis: age younger than 20 years, vascular purpura, acute abdominal pain, or leukocytoclastic cutaneous vasculitis. The presence of at least two of these signs demonstrates a sensitivity of 87.1% and a specificity of 87.7% [12]. In 1994, during the Chapel Hill Consensus Conference, the presence of IgA deposits in small cutaneous, intestinal, or renal vessels was added as a mandatory criterion.

Due to the involvement of small blood vessels throughout the body, this disease can cause a variety of symptoms in different organs [13], including the ureters and bladder (stenosing ureteritis, hemorrhagic cystitis, and hematomas are rare), testicles (in 9% of boys), central nervous system (in 2-7% of cases with multifactorial involvement), heart and pericardium (myocarditis and pericarditis), lungs and pleura (in less than 1% of cases), parotid glands (parotitis), hepatosplenomegaly, and cholecystitis. Although rare, these manifestations typically have a favorable prognosis. Severe involvement of soft tissues is seldom documented in the literature.

Compartment syndrome arises from an abnormal increase in pressure within a muscle compartment enclosed by an inflexible membrane. Consequently, vascular axes are compressed, leading to tissue hypoperfusion and potentially muscle ischemia, resulting in swelling and increased muscle volume. This elevates intramuscular pressure due to counter pressure from the inflexible walls. Capillary blood flow decreases, exacerbating ischemia, thus perpetuating a self-sustaining vicious cycle. Once initiated, this process becomes irreversible, prompting a race against time to disrupt the cycle.

Fasciotomy should ideally be performed within the first six hours, guided by clinical signs (pain and compartment tension) and compartment pressure measurements (with a differential pressure of <30 mmHg), aiming to mitigate potentially severe functional sequelae, including the need for amputation. Medico-legal implications are not uncommon.

According to the findings of Wang et al. [14], soft tissue swelling was noted in 30% of the 50 patients diagnosed with rheumatoid purpura, and Somekh et al. [15] noted that three children with HSP in their series developed severe symmetrical muscle pain in their legs, suggesting that intramuscular bleeding could be the underlying cause of their symptoms. It is therefore possible that intra-compartmental volume is increased by edema and hemorrhage resulting from HSP, which is the case in our patient.

To our knowledge, only two cases of compartment syndrome complicating HSP have been reported in the literature. However, in one case, the child also had a severe deficiency in factor XIII and was effectively treated with recombinant factor VIIa and fasciotomy. In this instance, compartment syndrome was attributed to a hematoma resulting from the severe factor XIII deficiency [16]. Although the exact mechanism of decreased factor XIII activity in rheumatoid purpura is not yet clear, one hypothesis suggests that factor XIII may be degraded by infiltrating leukocyte proteases or excessively consumed during fibrin formation around affected vessels. In the second case, compartment syndrome developed following the removal of an arterial line. Luis and Ng [17] suggested that three primary factors significantly contribute to the disease process: 1) activation of the complement pathway due to arterial cannulation breaching the endothelium; 2) lymphokines such as TNF and IL-1, which activate both the intrinsic and extrinsic coagulation pathways while reducing fibrinolytic activity, leading to vessel thrombosis; and 3) hemodynamic factors, including turbulence, ischemia, and increased venous pressure. They concluded that arterial cannulation should be avoided in children with HSP due to the potential risks of thrombogenic tendency, ischemia, soft-tissue edema, and intramuscular bleeding, all of which could lead to compartment syndrome.

In the same context, two cases of compartment syndrome were also reported in the setting of infantile hemorrhagic edema [18].

Another interesting aspect of our case is the severity of the clinical presentation and the occurrence of multiple complications. Identifying risk factors for organ and system involvement early can help prevent complications. Risk factors of renal involvement in HSP have been well detailed in literature and they include age between >10 years, male gender, high blood pressure, gastrointestinal bleeding, recurrent or persistent purpura, WBC > 15 × 109 /L , platelets > 500 × 109/L, decreased complement 3, hyperuricemia, anemia, and hypoalbuminemia [19]. As for gastrointestinal involvement, the most important risk factor is the high neutrophil/lymphocyte ratio [20].

Despite extensive research into the epidemiology, clinical features, treatments, and prognosis of HSP over many years, the question that persists is, why do certain children experience severe complications? Therefore, we believe that further investigation into genetic susceptibility to HSP, immune pathogenesis, and the development of more effective treatments is still warranted in this field.

## Conclusions

In the vast majority of cases, HSP is a benign condition. The incidence of atypical complications is not high, but some are fatal. Compartment syndrome during RP is exceptional, requiring early and tailored management to preserve functional prognosis. The frequency of traumatic etiology should not overshadow other causes of compartment syndrome.
